# Supplementary material for: Tailoring interfacial effect in multilayers with Dzyaloshinskii–Moriya interaction by helium ion irradiation
Source: Sci Rep. 2021 Dec 8;11:23626. doi: 10.1038/s41598-021-02902-y (PMC8654828; doi:10.1038/s41598-021-02902-y)
Supplement: Supplementary file 1 — Supplementary Information. [file 41598_2021_2902_MOESM1_ESM.pdf]

Supplementary Information for "Tailoring interfacial effect in multilayers with Dzyaloshinskii-Moriya interaction by helium ion irradiation"

A. Sud[1], S. Tacchi[2], D. Sagkovits[1,3], C. Barton[3], M. Sall[4], L. H. Diez[5], E. Stylianidis[1], N. Smith[3], L. Wright[3], S. Zhang[6], X. Zhang[6], D. Ravelosona[4,5], G. Carlotti[7], H. Kurebayashi[1], O. Kazakova[3], and M. Cubukcu[1,3,*]

[1]London Centre for Nanotechnology, University College London, 17-19 Gordon Street, London, WC1H 0AH, United Kingdom
[2]Istituto Officina dei Materiali del CNR (CNR-IOM), Sede Secondaria di Perugia, c/o Dipartimento di Fisica e Geologia, Università di Perugia, I-06123 Perugia, Italy
[3]National Physical Laboratory, Teddington, United Kingdom
[4]Spin-Ion Technologies, Palaiseau, France
[5]Centre de Nanosciences et de Nanotechnologies, Orsay, Île-de-France, France
[6]King Abdullah University of Science and Technology Physical Sciences and Engineering Division, Thuwal, Makkah, Saudi Arabia
[7]Dipartimento di Fisica e Geologia, Università di Perugia, Via Pascoli, I-06123, Perugia, Italy
[*]murat.cubukcu@npl.co.uk

S1 Derivation of FMR fit curves:

For FMR spectras, the resonance field (H_{res}) and the half-width-at-half-maximum linewidth ($\mu_0\Delta H$) of the FMR signal are determined by a fit using differential forms of symmetric and anti-symmetric Lorentzian functions, are given by equation S1 (S1):

$$\frac{dP}{dH_{DC}} = -V_{sym}h_{ac}\frac{2(H-H_{res})\Delta H^2}{(H-H_{res})^2+\Delta H^2)^2} - V_{asym}h_{ac}\frac{\Delta H((H-H_{res})^2-\Delta H^2)}{(H-H_{res})^2+\Delta H^2)^2}, \tag{S1}$$

Here, V_{symm} and V_{asym} denote the symmetric and antisymmetric Lorentzian components, where h_{ac} is the amplitude of ac magnetic field modulation.

S2 Resistivity measurements:

In Fig.4(e) of the main manuscript, we show the dependence of damping as a function of the IR. He^+ irradiation shows a profound effect on the relaxation properties as demonstrated by a non-monotonic increase in damping value α. The effect of irradiation on damping in our samples indicates that the change in damping with IR is due to a change in resistivity of the multilayers. The dominant magnetization relaxation in our films involves electron scattering. To quantify the effect of scattering as the dominant mechanism we calculated the resistivity of the multilayers, as shown in Fig.S1(a). Resistivity ρ is related to scattering rate τ as $\rho^{-1} = ne^2\tau/m^*$ and the damping α can be expressed as (S2):

$$\alpha \simeq \frac{\zeta\gamma m^* k_F}{2\pi^2 M\tau} \tag{S2}$$

Here, m^* is the effective mass of s-electron, k_f is the Fermi wave vector and ζ is a constant which is related to spin-relaxation rate τ_s by $\tau_s^{-1} \sim \zeta\tau^{-1}$. Using the Drude conductivity formula the expression calculated for 3d metals is given as (S2):

$$\frac{\alpha}{\zeta\rho} \simeq \frac{e^2 k_F}{\pi^2\hbar} \simeq 3.7\times10^5(\Omega m)^{-1} \tag{S3}$$

We see a correlation between resistivity and damping values, both showing an increase with increasing IR. The changes in ρ reflect the corresponding changes in α. By fitting the Fig.S1(b) with a straight line we obtain the value of $\zeta \sim 0.4$ i.e $\tau_s^{-1} \sim 0.4\tau^{-1}$. The results signify a large contribution of electron scattering on the relaxation properties of He^+ irradiated multilayers.

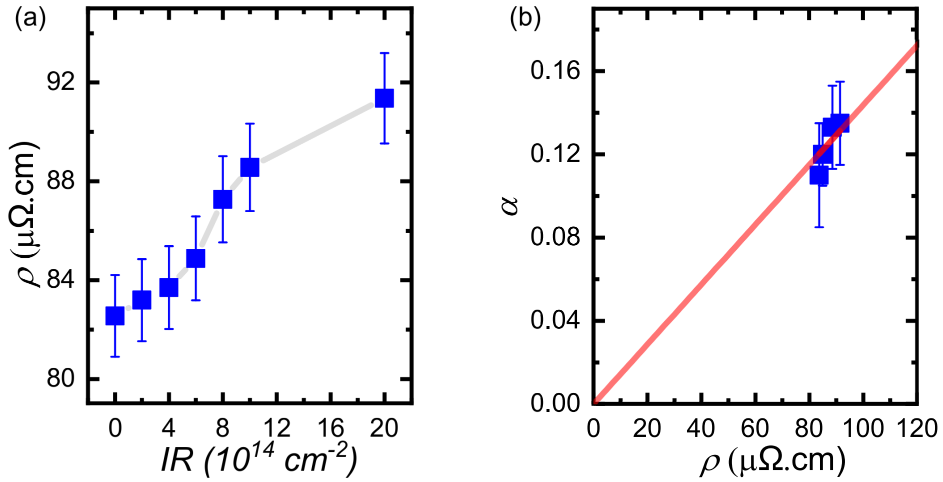

Figure S1. (a) Resistivity ρ as a function of IR (b) Damping constant α vs resistivity ρ.

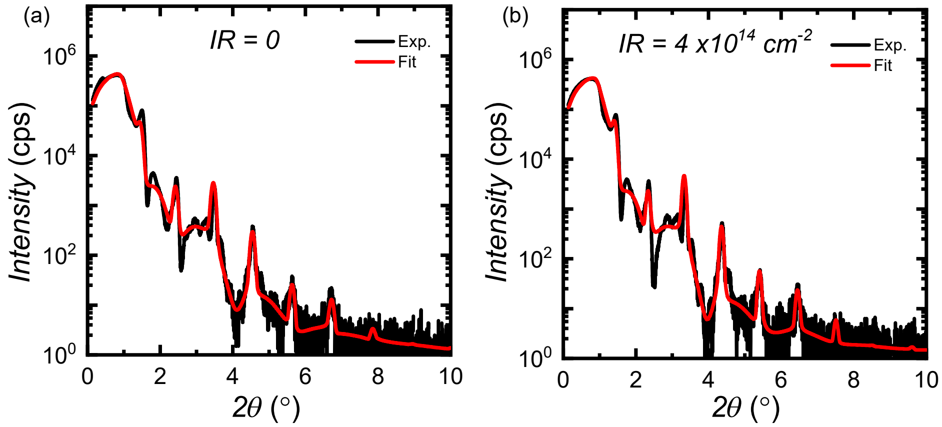

Figure S2. Intensity as a function of 2θ incident angle (a) $IR = 0$, (b) $IR = 4 \times 10^{14} cm^{-2}$.

S3 X-ray reflectivity measurements:

In Fig.S2, we show the X-ray reflectivity measurement results. Estimated fitting parameters shown in table 1.

S4 MFM measurements:

To demonstrate the ability to nucleate skyrmions in these materials a skyrmion lattice was nucleated using a magnetic force microscopy (MFM) tip and consecutively scanning the sample on our multilayer systems (S3) (Fig.S3). The images shown are taken from the as-grown sample without He^+ irradiation and under different applied magnetic fields Fig.S3(a-b). In Fig.S3(c) we show the skyrmions' area decreasing linearly with the applied field and saturate film at around 40 mT. The circularity is almost constant in all applied fields (inset).

Table 1. Fitting parameters for X-ray reflectivity measurements

Sample	Layer	$d(nm)$ thickness	$\sigma(nm)$ roughness
	Bottom Ta	4.3302 ± 0.2236	0.677 ± 0.289
	Pt	4.4383 ± 0.1089	0.540 ± 0.0837
	Co	1.1034 ± 0.0801	0.368 ± 0.049
$IR = 0$	Ta	2.4079 ± 0.1232	0.373 ± 0.0612
	Top Pt	4.4593 ± 0.0604	0.483 ± 0.0636
	Top Co	1.2062 ± 0.1639	0.318 ± 0.168
	Top Ta	1.7258 ± 0.2887	0.999 ± 0.074
	Bottom Ta	4.699 ± 0.153	0.687 ± 0.347
	Pt	4.602 ± 0.066	0.486 ± 0.072
	Co	1.218 ± 0.086	0.352 ± 0.064
$IR = 4 \times 10^{14} cm^{-2}$	Ta	2.481 ± 0.043	0.34 ± 0.059
	Top Pt	4.565 ± 0.082	0.467 ± 0.051
	Top Co	1.430 ± 0.115	0.528 ± 0.103
	Top Ta	1.422 ± 0.144	0.999 ± 0.098

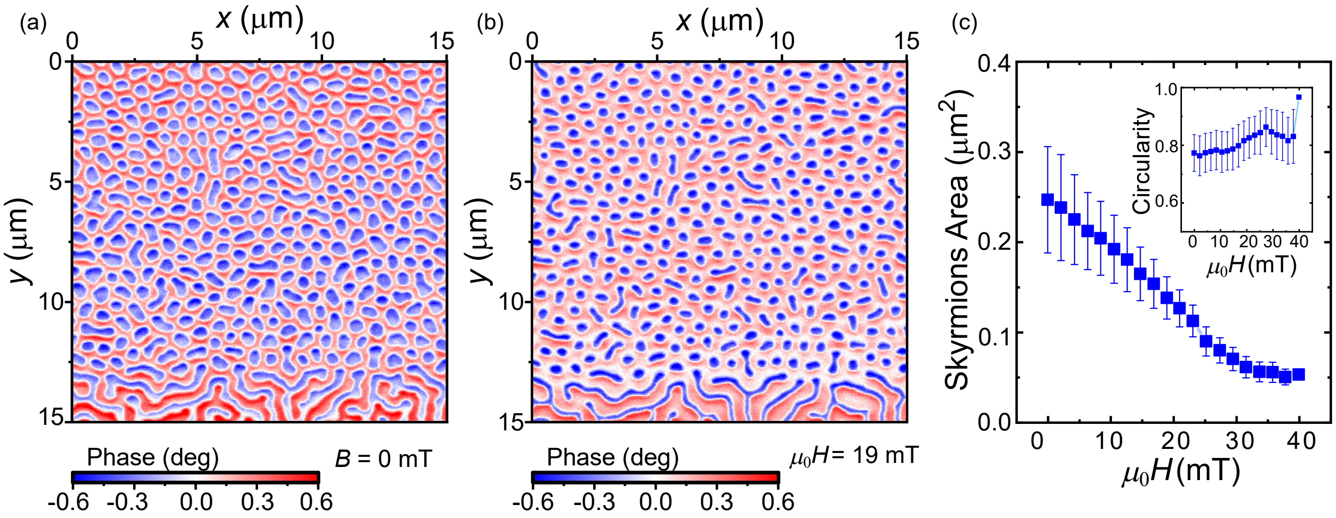

Figure S3. (a-b) MFM images on as-grown sample with different perpendicular applied magnetic fields $\mu_0 H$. The domains at the bottom of the images show the initial magnetic state of the samples which then we modified by using the MFM tip and an appropriate scanning protocol. A plot showing the area of the skyrmions (c) and circularity (inset) vs the perpendicular applied magnetic field $\mu_0 H$.

References

[S1] Rogdakis, K. *et al.* Spin transport parameters of NbN thin films characterized by spin pumping experiments. *Phys. Rev. Mater.* **3**, 014406 (2019).

[S2] Ingvarsson, S. *et al.* Role of electron scattering in the magnetization relaxation of thin $Ni_{81}Fe_{19}$ films. *Phys. Rev. B* **66**, 214416 (2002).

[S3] Zhang, S. *et al.* Direct writing of room temperature and zero field skyrmion lattices by a scanning local magnetic field. *Appl. Phys. Lett.* **112**, 132405 (2018).
